# The Impact of Taxes on Competition for CEOs

**DOI:** 10.1080/09638180.2016.1200477

**Published:** 2017-07-11

**Authors:** Peter Krenn

**Affiliations:** ^a^ Center for Accounting Research, University of Graz, Graz, Austria

## Abstract

This paper contributes to the question of how taxation of corporate profits and wages affects competition among firms for highly skilled human resources such as CEOs. Use of a theoretical model shows that wage taxes can have a substantial impact on the outcome of such a competition if marginal tax rates are different as in an international labor market. Further, the paper shows that increasing the wage tax rate unilaterally can have an ambiguous effect on observed gross compensation levels. However, in a local labor market for CEOs, observed gross fixed salaries should decline in the wage tax rate. Tax effects in a market for CEOs is a particularly interesting topic because recent developments with respect to compensation practices of top-level managers have opened a public debate about the use of instruments for regulating compensation of those managers. Furthermore, many countries around the world use tax incentives in order to facilitate immigration of highly skilled human resources. The investigation follows an analytical economics-based approach by extending an LEN model with elements of competition for scarce human resources and income taxation. It investigates the impact of differential taxation on the competition between two firms for the exclusive service of a unique, highly skilled CEO.

## 1. Introduction

Decisions of CEOs can have a fundamental impact on the financial success of firms. For this reason, many companies seem to exert an extraordinary high level of effort on the hiring decision of their top level management, and try to attract the most talented person for this important job by offering wage payments which appear to be far beyond the compensation levels of the existing management (e.g. Frydman and Jenter, 2010). For instance, in the US the CEO-to-worker compensation increased from 20-to-1 in 1965 to 510.7-to-1 in 2013 with an average CEO pay of $24.8 million (Mishel and Davis, 2014).^1^ Similarly, in 2014 the average CEO pay disclosed by UK FTSE 100 firms was 125 times the average employee pay pointing to a mismatch regarding compensation levels (Marsland, 2015). In trying to find valid explanations for the trend of increased CEO compensation, various studies suggest that firms are in competition for scarce human resources, including talented CEOs. The intensity and outcome of this competition are determined by various factors including firm sizes and the abilities of the CEOs involved (e.g. Gabaix and Landier, 2008; Terviö, 2008).

However, recent developments of compensation practices of top level managers have not only stimulated academic research, but also opened an ongoing public debate about these issues. In a world of growing inequality, this discussion has raised questions such as whether and how to regulate payments made by firms to their CEOs, or if there is any necessity to redistribute income from these top earners to the rest of the society. It appears that in the last years many governments around the world have begun to show their response by using the tax system to perform regulating and redistributive effects in the context of management compensation. For instance, in 2013 the Austrian government introduced a progressive tax system for bonus payments which formerly have been subject to a very generous preferential tax treatment. A major tax reform enacted in 2016 included a temporary increase in the marginal tax rate for people who earn more than €1 million a year. However, as labor markets (especially within the European Union) became increasingly integrated, and due to the fact that highly skilled people are comparably mobile, they are also able to respond to undesirable changes in their environment by migrating to other countries. Several studies show that highly skilled people's mobility responds to tax incentives (Kleven, Landais, and Saez, 2013; Kleven, Landais, Saez, and Schultz, 2014). The introduction of a 75% ‘super-tax’ in France serves as an anecdotal example how top earners react to changes in tax incentives. The tax was announced in 2012 and there were news about people who decided to emigrate or at least planned to do so including the famous actor Gerard Depardieu and the CEO of LVMH, Bernard Arnault. Hopkins (2014, December 23) indicates that the tax was abandoned in 2015 not only due to its low incidence but also due to difficulties of French companies to attract top international staff.

Taking into account these observations, governments seem to be confronted with the problem that individual income taxes in the shape of wage taxes do not only impact compensation contracts between firms and CEOs but can also have a substantial influence on the ability of firms within a country to attract highly skilled people. The effects of wage taxes are twofold in a market for talented CEOs. On the one hand, these taxes drive a wedge between the payment the firm offers the CEO and the payment this manager finally receives. As a direct effect they therefore tend to create the need for a higher gross compensation in order to preserve working incentives. On the other hand, wage taxes can also have an indirect effect on compensation levels and the competition for CEOs since they may affect all competing firms differently (e.g. due to existing pre-tax distinctions like CEO compatibility or different wage tax rates). However, until now our knowledge about how these effects work together in a market for talented CEOs is still limited. Therefore, the main contribution of this paper is to join the fields of managerial incentive design, competition for talents, and taxation and show how taxes impact the firms' competition for CEOs. Theoretical literature explaining the observed increase of CEO compensation by using a market model has not considered issues of international taxation. However, including differential taxes into a competitive model seems important because they directly affect offered compensation levels and are therefore able to distort the competition outcome. Further, the literature investigating effects of taxes on managerial incentives and compensation did not investigate so far the impact of taxes on the manager's alternative working opportunity that creates the reservation utility commonly used. Extending this theory by means of competition helps to show the overall effect of taxation on compensation levels because it captures the tax effect on the CEO's reservation utility in a consistent way. The paper examines the following research questions: How does taxation of corporate profits and wages affect compensation offers of firms that are in national and international competition for a highly skilled CEO? How do expected utility levels of CEOs and expected profits of firms change under taxation of corporate profits and wages? How does differential wage taxation impact a firm's ability to attract a highly skilled CEO?

The research questions are addressed by using an LEN model with two identical risk neutral firms (principals) competing with one another in order to hire a single CEO (agent). This CEO represents an effort averse ‘superstar manager’ with a unique ability to increase the expected profit of the hiring firm by performing a non-observable task.^2^ As this makes the manager desirable for both firms, each of them offers a compensation contract that (i) uses incentive pay in order to ensure a desired effort level in case the CEO is hired and (ii) tries to outperform the contract offered by the competitor and hence attract the CEO. The firm that fails to hire this superstar CEO has to hire an ordinary manager with no special ability and extra benefits. In this model the expected utility the CEO can receive from an agreement with either firm crucially depends on the obligated amount of wage tax to pay, and the compatibility of the CEO with the competing firms. The highly skilled manager's compatibility can be different among the firms due to various reasons including the match between firm size and ability, different organizational characteristics and culture, general economic conditions or being an internal candidate for one of the firms. Further, wage taxes might vary substantially among the firms because in an international setting the firms may be located in different tax jurisdictions with different tax rates. In order to present the impact of differential taxation within this setting in a meaningful way, the paper compares the situation of different tax rates applied at both firms to the situation where all contracting parties are subject to a uniform linear wage and corporate tax.

A central finding of this paper is that wage taxes become an important determinant of the competition outcome if wages are taxed at different rates. The paper illustrates that a sufficiently large tax rate differential is able to offset an existing pre-tax disadvantage a firm might have due to a lower compatibility with the manager. This leads to the result that the competition outcome can differ between uniform and differential taxation and indicates that countries should consider potential negative consequences when unilaterally increasing wage tax rates. However, the paper also shows that the tax effect can be outweighed by an additional risk effect in a situation of non-observable effort. This effect restores the competition outcome that would occur under uniform taxation if the tax advantage of the firm with lower compatibility is within an intermediate range and risk is sufficiently high and/or important. Additionally, under differential taxation, a marginal increase of the wage tax rate applied at the successful firm has an ambiguous effect on the offered fixed salary. This result also complements mixed empirical evidence about the effect of taxes on CEO compensation.

In contrast to these observations, one can find quite unexpected effects in a setting of uniform taxation. An increase of the uniform wage tax rate reduces the fixed salary offered by both competitors. This paradoxical effect occurs because wage taxes do not only reduce the expected utility that the CEO can gain from working for one firm, but also reduce the reservation utility which would be generated at the other firm. This in turn tends to lower the compensation needed by the successful firm in order to attract the CEO. The paper shows that this indirect effect of wage taxes on gross compensation dominates the direct tax effect, and hence observed fixed salaries decline under uniform taxation. Therefore, the paper indicates that assumptions concerning the taxation of an agent's reservation utility can be crucial when assessing the effects of taxes in agency models. By explicitly modeling the agent's alternative working opportunity, a possible way to address this issue is presented.

The paper proceeds as follows. Section 2 provides the reader with necessary background from prior literature and highlights the incremental contribution of the paper. Further, it presents details on underlying theory and the institutional setting. Section 3 explains the basic model setup. In Section 4 results for a competition among firms in a situation of observable and non-observable effort are derived. Additionally this section outlines the impact of marginal changes in tax rates. Finally, Section 5 concludes and discusses possible limitations of this investigation.

## 2. Background

### 2.1. Related Literature and Contribution

This paper contributes to and combines two nearly unconnected major streams of literature: First, it extends studies that explain recent trends in CEO compensation practices and in particular the increase of total CEO pay. Second, it advances research on the impact of taxation on the size and structure of executive compensation.

Total CEO pay has substantially increased over the last decades (e.g. Conyon, 2006; Frydman and Jenter, 2010; Murphy and Zabojnik, 2004) whereas wages of other workers have not experienced a comparable upward shift over the same period of time. Researchers have recognized this and other trends in CEO compensation and made efforts in developing theories that are able to explain the observed increases of compensation levels.^3^ One of those explanatory theories that has been well-established in recent years is based on specifics of the job market for CEOs. A CEO is considered to be a highly skilled and scarce human resource who has an important impact on firm performance. Therefore, firms are in competition for the most appropriate candidate and the severity of this competition is a fundamental determinant of pay level. In line with this argument, firms' increased demand for general management skills in recent years is consistent with the observation of more outside hires. This leads to more severe competition and hence high compensation levels (Murphy and Zabojnik, 2004, 2007). In a partial-equilibrium model, both papers depict firms' trade-off between promoting an internal and hiring an external CEO candidate when wages are determined in a competitive market. On one hand, hiring an outsider comes with the opportunity of a better fit between the ability needed by the firm and the ability offered by the CEO. On the other hand, it causes a loss of firm-specific knowledge which is available by the internal candidate only. A shift of demand from firm-specific skills to more general skills makes hiring an external candidate more attractive for the firms and therefore has a stimulating effect on competition and compensation levels.

Another group of researchers analyzed the job market for CEOs and determinants of CEO compensation levels in a more general setting by using an assignment model approach. This type of model describes how firms of different sizes and CEOs with different abilities are assigned in equilibrium if firm size and CEO ability are complements with respect to production output and the labor market is competitive. The base model ignores incentive problems and shows that the most talented CEO is hired by the biggest firm and all other market participants are matched by descending size and ability (Gabaix and Landier, 2008; Terviö, 2008). Further, the model predicts a positive association between total CEO compensation and firm size. Based on these findings, assignment models have also been used in order to study the structure of pay of talented CEOs. Edmans, Gabaix, and Landier (2009) develop a model which combines the assignment model equilibrium with an incentive problem. If firm value depends on both the CEO's ability and some privately known and costly effort, firms pay a fraction of total CEO pay in shares. However, the paper shows that the high levels in total pay are driven by the firms' competition for CEOs, and are not affected by the firms' problem to provide working incentives. Further, the low level of fractional ownership observed in practice is consistent with optimal contracting if a multiplicative specification of the CEO's utility function is used. The impact of risk has also been investigated in the CEO market equilibrium. If firms differ in size, risk, and the level of effort required, the matching with risk-averse CEOs becomes distorted (Edmans and Gabaix, 2011). Combining firm size, risk, and effort disutility into a single measure for assignment shows that providing incentives for high ability CEOs is more expensive and associated with the demand for a higher risk premium. Therefore, the agency problem causes high risk firms to prefer less talented CEOs and pay them higher salaries compared to their ability.

The theories about recent trends in CEO compensation have also been tested in several empirical studies. Terviö (2008) and Gabaix and Landier (2008) confirm the theoretical prediction of a positive association between CEO compensation and firm size. Moreover, Gabaix and Landier (2008) show that a sixfold increase in CEO compensation in the US between 1980 and 2003 can be explained by the same increase in market values during the same time. However, more recent studies challenge the explanatory power of a single theory on its own. Frydman and Saks (2010) analyze the long-run development of executive compensation in the US from 1936 to 2005 and find that a strong correlation between CEO compensation and firm size is limited to years after 1976. Further, their results suggest that the causal relationship between compensation and firm size is only limited. Based on the results of Gabaix and Landier (2008), Cremers and Grinstein (2014) hypothesize that the association between CEO compensation and firm size should be stronger in industries with a high demand for general management skills due to stronger bargaining power of CEOs. The demand for general management skills is measured as the relative amount of outside hires in each industry by using data for largest public US corporations between 1993 and 2005. They find only weak evidence for differences between ‘insider’ and ‘outsider’ industries and conclude that the market theory for CEOs is not able to explain observed high compensation levels fully.

The role of income taxation on executive compensation has been addressed within both, theoretical and empirical tax research. So far, theoretical contributions have based their investigations on different types of agency models without considering forces of competition on the job market for CEOs. A well-researched stream of literature describes the impact of linear income taxes on the contractual relationship between firm (principal) and manager (agent) if the manager is privately informed about the effort choice (moral hazard). In general, linear corporate taxes have no impact on the agent's effort or contractual incentives if the manager's expected utility is unaffected by corporate taxation. For instance, Katuscak (2004) presents this result within a general model of continuous effort and continuous share prices where the agent's absolute risk aversion is constant. Further, Niemann (2008) includes taxes into an LEN model with portfolio choice and shows that firms fully filter out distortions induced by corporate taxes. However, making fixed compensation only partially tax-deductible results in an increase of variable compensation and higher managerial effort. This has been illustrated by  Halperin, Kwon, and Rhoades-Catanach (2001) who investigate the impact of Section 162(m) of the Internal Revenue Code within a moral hazard model of binary income and continuous effort.^4^ Göx (2008) extends an LEN model with a random factor beyond the manager's control and shows that the deductibility limits on fixed compensation may even trigger firms to reward for luck.

In contrast to corporate taxes, wage taxes have a direct effect on the design of compensation contracts. Wage taxes reduce the agent's remuneration directly and therefore the compensation that the principal has to pay normally differs from the amount of money the agent receives. This ‘tax wedge’ leads to three important theoretical results: First, wage taxes can have a negative effect on the effort exerted by the agent as compensating for the same amount of effort becomes more expensive (Katuscak, 2004; Niemann, 2008). Second, wage taxes tend to increase fixed compensation as well as total compensation if the agent's reservation utility stays unaffected by taxation (e.g. Halperin et al., 2001). Finally, the effect of wage taxes on variable compensation is ambiguous and depends on the underlying model (Katuscak, 2004). Where Niemann (2008) finds for an LEN model that pay-performance sensitivity is unaffected, Halperin et al. (2001) document that the bonus for success increases in the wage tax rate. The impact of international differences in income taxation on the contract design for managers has been so far researched only to a very limited extent. Martini and Niemann (2015) investigate the impact of differential taxation on assignment decisions of human resources under different methods for avoiding double taxation using an LEN model. They find that the optimal assignment decision depends on both corporate and wage taxation. Further, the impact of the variation in the wage tax rate on the optimal assignment decision is ambiguous and depends on the method for avoiding double taxation.

Despite the mostly clear theoretical predictions, empirical evidence about the effects of taxation on the size and composition of executive compensation is mixed. Hall and Liebman (2000) investigate whether changes in tax policy had a substantial impact on the increased use of stock-option grants. They find no evidence that a preferential tax treatment of options triggered the observed changes in the composition of pay. Further, they document that there has been only a minor substitution between fixed and performance-related pay following the introduction of a deductibility limit for fixed salaries. However, Katuscak (2009) provides empirical evidence for a decrease of pre-tax pay-to-stock-price sensitivity generated by stock option grants following an increase in the top marginal wage tax rate. Frydman and Molloy (2011) indicate that a negative association between executive compensation and wage taxes exists only in the long run. They find total compensation, as well as the structure of compensation, to be unresponsive to major changes in tax rates. However, all studies use a sample of US firms and therefore lack the ability to exploit another source of variation in wage tax rates, which occurs in an international labor market for CEOs.

The main contribution of this paper is to join the above-mentioned two streams of literature and to show to what extent taxes affect a firm's ability to hire a suitable CEO. Considering taxation as a potential distortion for the market for CEOs is important. This is because prior studies have shown that tax incentives can be used in order to attract highly skilled individuals and also have an effect on offered compensation levels. For instance, Kahanec and Zimmermann (2011) are able to show that many countries in Northern and Western Europe facilitate the attraction of a highly skilled workforce through their immigration policies by providing generous tax provisions to people who qualify as highly skilled. Kleven et al. (2013) analyze the European market for football players and show that player mobility responds to tax rates. Further, low tax rates can cause a replacement of low-ability players with high-ability players within a country. Kleven et al. (2014) investigate empirically the response of top earners to tax incentives which are granted in Denmark for facilitating international migration and find a significant increase of highly paid foreigners eligible to a preferential tax scheme. They show also that pre-tax income decreases with a decline of the average tax rate. Similarly, Ruf and Schmider (2015) find for executive compensation data from 28 countries that CEO gross compensation increases with top marginal tax rates. Both studies explain this effect with the bargaining power of highly skilled human resources.

The model used in this paper combines an agency model with a competitive hiring game between two firms and includes (differential) taxation. The paper differs from existing theoretical models describing the market for CEOs in three major ways. First, it includes taxation of corporate profits and wages which allows therefore an analysis of tax-induced distortions on CEO assignment. Second, instead of using firm size and CEO ability as prime determinants for assignment, the model implements a single measure of compatibility (‘fit’) between CEO and firm. This single parameter implements the combination of all relevant assignment characteristics and allows to focus on distortions generated by taxes. Finally, the paper does not derive a market equilibrium. Such an analysis is mainly omitted for two reasons. Firstly, assignment models become difficult to describe, solve and interpret, if the matched items differ in more than one characteristic each (see for instance Edmans and Gabaix, 2011). However, in this paper the involved contracting parties can differ in compatibility, corporate tax rates and wage tax rates. Secondly, the focus of this paper is to show to what extent taxation potentially generates distortions among firms. Therefore using a partial equilibrium model can be sufficient.

The model also differs from prior theoretical contributions investigating the impact of taxes on managerial incentives and compensation. These studies normally rely on simplifying assumptions regarding the impact of taxes on the manager's reservation utility (e.g. Niemann, 2008). However, the need to assume how taxes affect the manager's outside opportunity makes it difficult to investigate an overall tax effect on managerial behavior and compensation. Also it may be a possible reason why theoretical and empirical results do not match. The model in use makes the manager's outside opportunity endogenous by explicitly modeling a second firm (principal). This approach ensures that effects of taxes on the manager's reservation utility are captured in a consistent way via the second firm. Therefore, the market extension of the commonly used agency model allows to present overall effects of taxes on CEO gross compensation instead of results regarding only the ‘marginal tax incidence’ (e.g. Katuscak, 2004). As the model shows, this overall effect can be ambiguous and dependent on various factors including firm risk, CEO compatibility and tax rates. Therefore the model can also help to explain why empirical evidence about the impact of taxation on CEO compensation levels is mixed.

Besides contributing to the literature streams already mentioned, this paper also adds to accounting research investigating incentive design and the regulation of executive compensation. For instance,  Larcker, Ormazabal, and Taylor (2011) and Hitz and Müller-Bloch (2015) investigate market reactions to the regulation of executive compensation. Both studies find that stock markets in particular reacted negatively to regulation for firms providing abnormally high executive board remuneration. As a potential explanation for this unexpected empirical result they hypothesize that shareholders might perceive regulation as an obstacle for efficient contracting. Analyzing the market for CEOs with the model used in this paper can also help to explain this empirical result. Abstracting from differences in taxation the model predicts that a CEO becomes hired by the most compatible firm, because this firm can offer the highest expected utility. However, regulation of compensation restricts the firm's ability to compete with other firms in order to hire the highly skilled CEO. As a result, the CEO might get hired by another, less compatible firm and the loss of economic rents generated by the CEO translates into negative market reactions.

### 2.2. Theory and Institutional Setting

The investigated competition among firms for a highly skilled CEO requires that CEOs are mobile to some extent and that firms are actually trying to hire external candidates in reality. In an international setting, the CEO mobility has to occur along two different dimensions: across firms and across geographic regions. There exists a lot of anecdotal evidence about established companies hiring an external CEO in recent years (see for instance Karaevli and Zajak, 2012). During 2016 in Germany, the former CEO of Henkel, Kaspar Rorsted, was poached by Adidas (Jervell, 2016, January 18). Stock prices of both firms reacted quite strongly to the announcement of the move which serves as an indicator that shareholders cared about the CEO in place and believed in a link between CEO and firm performance. Empirical studies also find that a considerable part of newly hired CEOs are outsiders varying from 18% (Allgood and Farrell, 2003) to 28% (Ryan and Wang, 2012). CEO mobility across geographic regions is likely to differ around the world. Whereas it is quite persuasive that this type of mobility should exist within a homogeneous area like the US it is rather unclear if this is also the case for Europe. Even though the regulations within the European Union provide a fertile ground for integration of national labor markets, there still exist barriers such as differences in culture or language that could restrict CEO mobility. Unfortunately there has been little research about the international mobility of top executives in Europe. The consultant company ‘Robertson Associates’ provides some weak evidence for Central Europe that managers in this region are mobile (Robertson Associates, 2015). Van Veen and Marsman (2008) study the nationality diversity of executive boards of the largest firms in 15 EU member states and conclude that with an average of 14.9% the proportion of foreigners is quite low. However, there is a huge variation among different countries from 2.5% in Spain to 76.2% in Luxembourg. Further, the study does not investigate the nationality diversity of CEOs separately. Arnegger, Hofmann, Pull, and Vetter (2010) analyze the demographic structure of German supervisory boards and find that on average 13% of the board members are foreigners. In summary, mobility of CEOs across firms and geographic regions exits but it seems to be quite moderate. However, it should be noted that a moderate number of observed outside hires and international CEOs does not mean that a competition among firms does not exist as it is only possible to observe empirically firms that actually hire a CEO.

The paper complements executive succession literature suggesting that a good match between firm and CEO has a positive impact on firm performance (for recent reviews see Giambatista, Rowe, and Riaz, 2005; Kesner and Sebora, 1994). These studies have identified the origin of the CEO (insider vs. outsider) as a key determinant for a good match. However, whether an internal or external candidate fits better depends on the situation of the firm. For instance, insiders tend to be a better match if the prior CEO resigned whereas outsiders have a better fit and performance if they follow a dismissed CEO (Allgood and Farrell, 2003). Further, firms that experienced a poor stock price performance are more likely to hire external CEOs (Datta and Guthrie, 1994) and CEOs previously working for many employers (Ryan and Wang, 2012). In general, these ‘mobile’ CEOs appear to be more capable of initiating a change in the firm's strategy which makes them more suitable for poor performing firms. The general structure of the model used in this paper brings the advantage that it does not rely on an assumption about the origin of the CEO. Each of the hired CEOs can also be interpreted as an insider for one of the competing firms where the insider's fit depends on the prior economic situation of the firm.

Even though the paper has a focus on the hiring of a new CEO, the analysis yields also valid results for cases where the CEO does not change. Retaining instead of replacing an incumbent CEO can also be interpreted as ‘hiring’ this CEO in context of the model in use. Therefore, the paper captures at least three different settings: First, the firm has to actually hire a new CEO because the previous CEO is unavailable. Second, the firm has an incumbent (ordinary) CEO and wants to improve its performance by hiring the superstar CEO. Losing the competition and ‘hiring’ the ordinary CEO would then simply mean that the incumbent is retained. Third, the superstar CEO is already incumbent at the firm but threatens to leave. In this case the ‘hiring’ could be interpreted as a renegotiation of the original contract in order to retain the superstar CEO.

Finally, as the paper investigates the impact of differences in corporate and wage tax rates, a brief overview about the actual cross-sectional variation underlines the potential relevance of the chosen setting. Differences in tax rates are most likely to occur in an international competition between firms lying in different countries. Within the OECD, corporate tax rates differed from 8.5% (Switzerland) to 35% (US) in 2015. Similarly, the top statutory personal income tax rates were between 16% (Hungary) and 57% (Sweden) in the same year (OECD, 2016). However, tax rates may even differ within a single country leading to additional variation. For instance in 2013 in the US the individual income tax rate varied on a state level from 0% to 12.3% additional to national taxes. Other examples for divergent income taxes are the sovereign cantonal tax regimes in Switzerland or municipal income taxes in Denmark and Finland (Schellekens, 2013). Taking these observations together indicates that differences in corporate tax rates and wage tax rates can be significant.

## 3. Model Setup

In order to address the question how competition and contract design for talented CEOs are affected by taxation, the paper develops a partial equilibrium model that extends an LEN type agency model (e.g. Holmstrom and Milgrom, 1987; Spremann, 1987) with elements of competition for scarce workforce and taxes. In particular, it considers the situation of two firms (principals) which are in competition to hire a unique superstar CEO *S* (agent). However, the firm that fails to attract this manager has to hire an ordinary manager *O* from the remaining labor market. The superstar CEO is risk-averse and able to cause a positive impact on the realization of the uncertain cash flow of the firm, 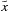, via a privately known effort *e*, whereas an ordinary manager has no special ability to increase this cash flow. The realized cash flow of firm 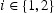 equals 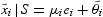 if the firm is able to hire the superstar manager and 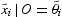 otherwise. The random noise term 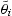 which is not observable for the firm, represents all kinds of uncertainty associated with the generation of the cash flow and follows a normal distribution with zero mean and variance 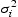. The random shocks affecting the cash flow of either firm 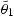 and 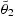 might be correlated with each other in any arbitrary direction. However, as the further analysis will show it is not necessary to make an assumption regarding their association because the respective cash flows can only be used separately within this model. For the ease of exposition it is assumed that both firms face the same business risk and hence 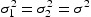. Further, the marginal impact of the agent's effort on the realized cash flow is equal for each firm with 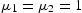. These assumptions can be justified especially for those cases where two firms of the same industry and with similar size are competing for the exclusive service of the CEO.

When firm *i* is able to hire the superstar manager *S* it has to pay a gross compensation of 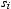. As the effort the CEO exerts can not be observed by either firm, each of them makes the offer of compensation dependent on the realized cash flow in order to provide working incentives for the agent. Hence, in this case the firm's gross profit equals 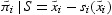. It is assumed that the ordinary manager's gross compensation equals a salary that is determined in a competitive market and is normalized to zero in this model. Therefore, hiring *O* would generate a profit equal to 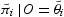.

As the paper aims to analyze competition among firms on a highly integrated international job market for scarce human resources it is assumed that both firms can be located in different tax jurisdictions with different tax systems. Such a market seems to be a plausible one for CEOs which are hired by very large multinational enterprises. However, the competitors do not necessarily have to be in different nations as there also exist many countries in the world with income tax rates varying locally among states, districts or municipalities. The taxable base for corporate taxation is uniform among firms and equal to the realized profit 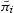. However, this profit is subject to a proportional corporate tax rate 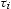 with 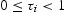 which can be different for both competing firms. Although different tax systems might imply differences in the tax rates and the taxable bases at the same time, the conjunction of these differences is approximated in this model by different tax rates only. Hence, all tax rates used in this paper could be interpreted as effective tax rates instead of nominal tax rates. Under the assumptions made, the realized after-tax profit of firm *i* is equal to 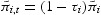.^5^

The CEO's utility depends on the after-tax compensation 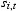 that can be received from firm *i* and the costs 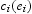 of exerting effort for this firm. The utility function of the CEO has an exponential form and is characterized as follows:
(1)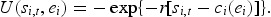 This type of utility function allows for explicit solutions with respect to the principals' maximization problem and has also been used for prior research on tax effects in agency models (e.g. Ewert and Niemann, 2012; Martini and Niemann, 2015; Niemann, 2008). The parameter *r*>0 represents the coefficient of constant absolute risk aversion of the agent. It is assumed that both firms offer only linear gross compensation contracts with:
(2)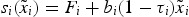 consisting of a fixed salary 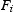 and a bonus coefficient 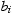.^6^ The CEO's realized gross compensation 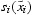 is subject to a proportional wage tax at the rate of 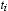 with 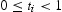. Therefore, the after-tax compensation is equal to 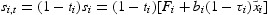.

The implementation of the wage tax as described above comes with two potential problems that should be discussed briefly here. First, assuming that the CEO's total compensation is subject to a single proportional wage tax rate ignores the possibility that different compensation parts can be taxed at different rates. In reality, however, a differential taxation can be the case when incentives are provided by the use of stocks or stock options, because these compensation parts commonly apply to capital gains taxation or are even tax exempt. Therefore, in these cases the wage tax rate 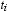 should be interpreted as an average weighted tax rate consisting of the country's top marginal tax rate on wages and the country's tax rate on capital gains. Second, due to the assumption of normally distributed cash flows the CEO's compensation might become negative if the realization of 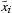 is sufficiently low. The model does not impose any limits on the CEO's liability and does not include any loss offset restrictions. Therefore, a negative compensation would lead to a full tax reimbursement to the CEO. Even though these assumptions appear to be quite contrary to what can be observed in the real world, there exist certain scenarios where negative compensation and tax reimbursements are effectively in place. For instance, a long-term compensation contract could include a provision of deferring and offsetting negative bonus payments with positive payments in subsequent years. This in turn would lower the total compensation and reduce the taxable income of the CEO in a later period and as a whole. An example for such a compensation practice can be found, for instance, in the annual financial report 2014 of the Austrian plant manufacturing company ‘Andritz AG’ (see Andritz, 2015).

For the purpose of interior solutions the agent's effort costs are considered to be quadratic with 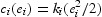. Hence, the cost function is in line with the generally accepted economic principle that marginal costs of personal effort are increasing. Without loss of generality it can be assumed that 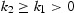 and therefore one extra unit of effort is more costly for the agent when providing it for firm 2 in comparison to firm 1. The different marginal cost parameters 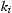 can be interpreted as different compatibility (higher values indicate lower ‘fit’) the CEO actually has for the competing firms which might be caused by various reasons. In line with prior literature on the market for talented CEOs, the compatibility parameter could be interpreted as a measure for the match between firm size and the CEO's inherent ability. A difference in organizational characteristics or culture within the firms might also serve as a potential explanation for different compatibility. This includes also characteristics regarding the top management team the CEO has to work with. Additionally, if both firms lie in quite different economic environments the CEO might require different amounts of effort to produce the same ‘output’. Finally, the difference in compatibility could indicate that the superstar CEO is an inside hire for one of the two firms.

Provided the assumptions of a normally distributed wage payment, and the negative exponential utility function with constant absolute risk aversion, the expected utility of the CEO can be expressed in terms of the corresponding certainty equivalent (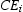):
(3)
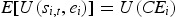

(4)
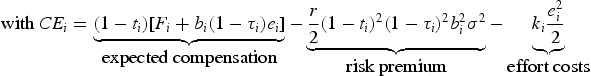



The certainty equivalent consists of the expected after-tax wage minus a risk premium and costs for exerting effort. The risk premium increases in the manager's risk aversion *r*, the bonus parameter 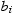 and variance of the uncertain cash flow 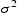. However, both tax rates have a moderating effect on the risk premium as they reduce the overall variance of the manager's compensation. It is assumed that both firms are able to offer a contract which the manager strictly prefers to any other outside option (including possible unemployment). Hence, the CEO decides to work for the firm which offers the higher certainty equivalent and randomizes with equal probability if both firms offer an equal amount. Put differently, from the perspective of contracting firm *i* the CEO demands a reservation utility which is represented by the certainty equivalent offered by rival firm *j* and vice versa.

As both firms are considered to be risk neutral they want to maximize their expected after-tax profits. Conditional on being able to hire the CEO the expected after-tax profit of firm *i* equals 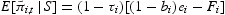. However, if firm *i* has to hire an ordinary manager the expected after-tax profit is equal to 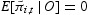. Provided the CEO's decision rule for selecting an employer, firm *i* can choose the certainty equivalent it wants to offer the agent strategically by setting 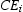 equal to an amount of 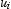. The ex-ante expected after-tax profit of firm *i* is then dependent on the strategies 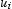 and 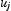 of both firms and has the following form:
(5)
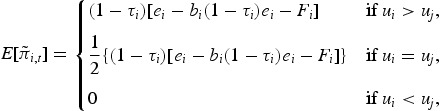

(6)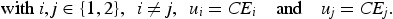 The first line in (5) indicates the case where firm *i* is able to hire the superstar CEO by offering a higher certainty equivalent than firm *j*. In the second line both firms offer an equal amount and therefore the manager is hired only with probability of one half. In the final case firm *i* loses the competition for the superstar and hires an ordinary manager.

**Figure 1.**
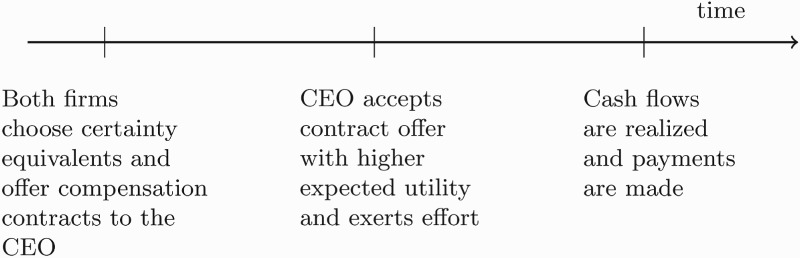
Timeline

To summarize, each firm faces two interrelated problems. First, it wants to provide sufficient working incentives in order to maximize its profit conditional on hiring the superstar manager (contracting problem). Second, it has to choose strategically a certainty equivalent offer which ensures that the manager is attracted (competition game). Figure 1 depicts the timing of the game. In stage 1 both principals simultaneously choose a certainty equivalent 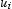 and 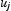 they want to offer the CEO and provide a contract offer 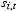 and 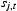. In stage 2 the CEO decides to work for one of the two firms and exerts effort. In the final stage the cash flows are realized, the CEO receives a compensation and taxes are paid.

## 4. Results

This section presents the results of the model for two different situations commonly considered in agency theory literature. Section 4.1 investigates a situation where the manager's effort is observable. Section 4.2 derives results for a situation of moral hazard where the manager has private information about the effort choice. In both sections the analysis follows the same structure. First, the contracting problem of each firm conditional on hiring the CEO is solved. In a second step, the optimal contract parameters are used in order to derive the outcome of the competition game for four different sub-settings. These sub-settings arise from the combination of different assumptions regarding the CEO's compatibility (equal vs. different compatibility) and taxation (uniform vs. differential taxation). Additionally, Section 4.3 presents the marginal impact of tax rate changes when effort is unobservable and compatibility as well as tax rates are different among firms.

### 4.1. Equilibrium with Observable Effort

The firms' optimal strategies and the outcome of the competition game depend on the expected profit that can be earned if the superstar CEO is hired. In order to calculate this expected profit the firms' contracting problem is solved in a first step. In the first-best case the effort exerted by the manager can be observed and verified by each firm. Even though this case occurs quite rarely in reality, it should serve as a benchmark in order to evaluate the consequences of private information on the competition game among firms. As each firm can directly control the amount of effort exerted by the CEO, no variable pay is needed in order to provide working incentives for the agent. For any given strategy in the competition game the contracting problem of firm *i* conditional on hiring the CEO is the following:
(7)
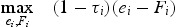

(8)
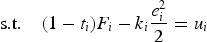
 In contrast to the standard agency problem 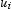 represents the strategy firm *i* chooses in the competition stage and not the agent's reservation utility (the certainty equivalent offered by the rival firm) per se. Therefore, the firm maximizes its expected after-tax profit by defining a level of 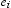 and 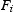 that provides the CEO exactly with the certainty equivalent strategy 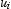. The optimal contract parameters can be calculated by using the Lagrangian approach and take the following values:^7^
(9)
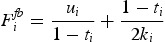

(10)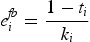 It is easy to observe that the corporate tax does not have any influence on the optimal contracting parameters whereas the wage tax has a direct proportionally negative impact on the contracted effort level. These results are in line with other papers investigating the role of taxes in a non-competition setting (e.g. Niemann, 2008).^8^ However, 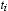 has two opposing direct effects on the fixed remuneration. On one hand, due to the reduction of exerted effort, it tends to reduce gross compensation. On the other hand, it creates the need for grossing up the offered amount of utility, because the tax reduces the net wage received by the agent. However, the overall effect of taxes on offered compensation levels cannot be evaluated within the contracting problem alone because 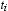 as well as the competitors' wage tax rate 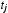 can have an impact on firm *i*'s optimal strategy 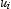 which is determined by the competition game. By using the parameters of the optimal contract it is possible to calculate the expected after-tax profit of firm *i* conditional on hiring the CEO:
(11)
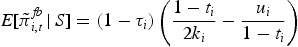



This expected profit can be used to derive the optimal strategy of each firm in the competition game. In order to provide a better understanding of how different situations of compatibility and taxation affect the competition for the CEO among the firms, results are derived for four different cases:

1. equal compatibility (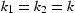) and uniform taxation (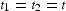 and 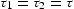)
2. different compatibility (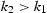) and uniform taxation (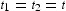 and 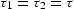)
3. equal compatibility (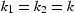) and differential taxation (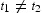 and 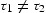)
4. different compatibility (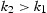) and differential taxation (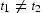 and 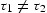)

Propositions 1– 4 present the equilibrium of the competition game for each of the four different cases. As the competition game is similar to a game describing a Bertrand market the derivation of the competition equilibrium can be done in line with literature on industrial organization (see for instance Shy, 1995; Tirole, 1988; Wolfstetter, 1999). The propositions show how the winner of the competition is determined and present the amount of certainty equivalent each firm chooses in equilibrium. The results for case 1 (equal compatibility and uniform taxation) are summarized in Proposition 1.^9^

Proposition 1. If the CEO is equally compatible with each firm and taxation is uniform, the competition winner is determined independently from compatibility and taxation. Both firms hire the CEO with equal probability by offering the same amount of certainty equivalent 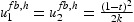. The expected profits of both firms are equal to zero.

Proof. See Appendix.

Proposition 1 shows that both firms are in a race to the top in order to attract the talented and therefore valuable CEO. Each firm is willing to sacrifice all the economic rents generated by the superstar and expected profits are driven to zero making each firm indifferent between hiring the superstar or an ordinary manager. As both firms face an identical situation with respect to compatibility and taxation, both end up in offering the same amount of certainty equivalent and the CEO becomes indifferent between working for firm 1 and firm 2. The competition outcome changes, however, if the firms differ in compatibility (case 2). This is indicated in the following Proposition 2.

Proposition 2. If the CEO is more compatible with firm 1 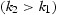 and taxation is uniform, the competition winner depends on compatibility only. Firm 1 always wins the competition and hires the superstar CEO. Both firms offer the same amount of certainty equivalent 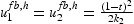. The expected profit of firm 1 is strictly positive whereas the expected profit of firm 2 equals zero.^10^

Proof. See Appendix.

Proposition 2 shows that the less compatible firm 2 behaves in the same way as with equal compatibility. It would be willing to give up any economic rent from hiring the agent and offers a certainty equivalent that yields an expected profit equal to zero. However, under different compatibility firm 2 fails to hire the CEO, because firm 1 is able to use its compatibility advantage. Everything else equal, a higher compatibility translates into a higher economic rent from working for firm 1. Firm 1 uses this potential and offers an amount that attracts the CEO with certainty. The equilibrium certainty equivalent offered by firm 1 yields the following expected profit:
(12)
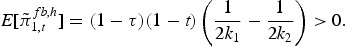

An inspection of Equation (12) reveals that the expected profit of firm 1 increases in the compatibility differential between the two firms. Even though uniform taxation has no impact on determining the competition winning firm, two important effects of taxes on equilibrium variables can be identified: First, the expected profit of the competition winning firm 1 decreases in the corporate tax rate and the wage tax rate. This can be seen from the multiple of 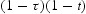 in Equation (12). Second, in equilibrium uniform wage taxes lower the amounts of certainty equivalent offered by both competing firms. As these amounts represent the expected utility that the CEO receives in equilibrium, it can be concluded that a uniform wage tax has a negative impact on the CEO's reservation utility.

The certainty equivalents, offered by each firm in equilibrium can not be observed in reality. Therefore it is particularly interesting, how these values translate into offered compensation levels, derived from the contracting problem. The equilibrium values of the offered fixed salaries can be calculated by inserting the optimal certainty equivalents into (9):
(13)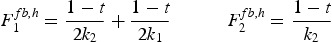 The compensation offered by firm 1 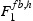 consists of two different parts. Whereas the first part compensates the CEO for opportunity costs (the certainty equivalent the CEO would earn when working for firm 2) the second part compensates for the amount of effort that has to be exerted when working for firm 1. The overall effect of uniform wage taxes on offered compensation levels follows from first derivatives of (13) and is presented in Corollary 1:

Corollary 1. In a situation of uniform taxation and observable effort, wage taxes reduce the gross salaries offered by both competing firms.

Proof. See Appendix.

The result of Corollary 1 appears to contradict prior findings of theoretical literature about tax effects on managerial compensation (e.g. Halperin et al., 2001) and therefore calls for further explanation. If both firms are in competition, the CEO has the power to extract all economic rents which (hypothetically) could be generated by working for firm 2, leaving firm 2 with an expected profit equal to zero. In this situation, an increasing wage tax rate reduces these economic rents due to its negative effect on the CEO's optimal effort level as indicated in Equation (10). This effect, however, exerts pressure on firm 2 to reduce the offered gross salary in order avoid a negative profit. As a result, the reduction of expected utility the CEO could receive from firm 2 has also a softening effect on the competition between the two firms and enables firm 1 to reduce the offered gross salary as a response. Further, the wage tax also reduces the level of effort the CEO actually exerts when working for firm 1. This has an additional negative effect on the compensation offered by firm 1.

The results in Propositions 1 and 2 illustrate that uniform taxation has no impact on the question of which firm wins the competition and hires the CEO. The remaining part of this section explicitly allows for different tax rates among the firms. This can be considered as a situation of international competition. Proposition 3 starts with the case of equal compatibility (case 3) and summarizes the equilibria and outcome of the competition game.^11^

Proposition 3. If the CEO is equally compatible with each firm and taxation is differential, the competition winner depends on the relation between the wage tax rates 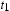 and 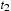 only.

1. If 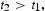 firm 1 wins the competition and hires the superstar CEO. Both firms offer the same amount of certainty equivalent 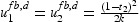. The expected profit of firm 1 is strictly positive whereas the expected profit of firm 2 equals zero.
2. If 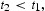 firm 2 wins the competition and hires the superstar CEO. Both firms offer the same amount of certainty equivalent 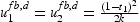. The expected profit of firm 1 equals zero whereas the expected profit of firm 2 is strictly positive.^12^

Differential taxation at CEO level can have a substantial impact on the competition among firms. In the case of equal compatibility, the different wage tax rates become the only force determining the outcome of the competition by providing the firm with the lower tax rate competitive advantage. Further, it should be noted that the certainty equivalent offered by the competition winning firm is only dependent on the wage tax rate applied at the inferior firm. As long as the relation between both tax rates holds, the ‘local’ wage tax rate has no impact on the offer of the successful firm, because this firm has only to slightly overbid the offer of the rival firm. Proposition 3 shows also that under differential taxation, corporate taxes have no influence on the competition for CEOs. As 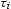 simply scales the expected profits of either firm it is irrelevant for the firms' decision on which utility level to provide to the CEO.^13^ Finally, Proposition 4 presents the results for a situation where the firms can differ in both, compatibility and taxation (case 4).

Proposition 4. If the CEO is more compatible with firm 1 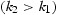 and taxation is differential, the competition winner depends on the relation between the compatibility parameters and the wage tax rates 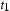 and 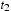. There exists a unique cut-off value 
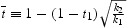
 that is smaller than 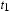 and can be used to determine the competition winner.

1. If 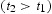 or 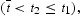 firm 1 wins the competition and hires the superstar CEO. Both firms offer the same amount of certainty equivalent 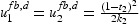. The expected profit of firm 1 is strictly positive whereas the expected profit of firm 2 equals zero.
2. If 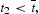 firm 2 wins the competition and hires the superstar CEO. Both firms offer the same amount of certainty equivalent 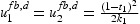. The expected profit of firm 1 equals zero whereas the expected profit of firm 2 is strictly positive.^14^

The impact of different wage tax rates becomes more subtle, if both firms differ additionally in compatibility. In this case, differences in tax rates can enforce, weaken or even outweigh the compatibility differential. The competition winner depends on the net effect of the two different forces.

**Figure 2.**
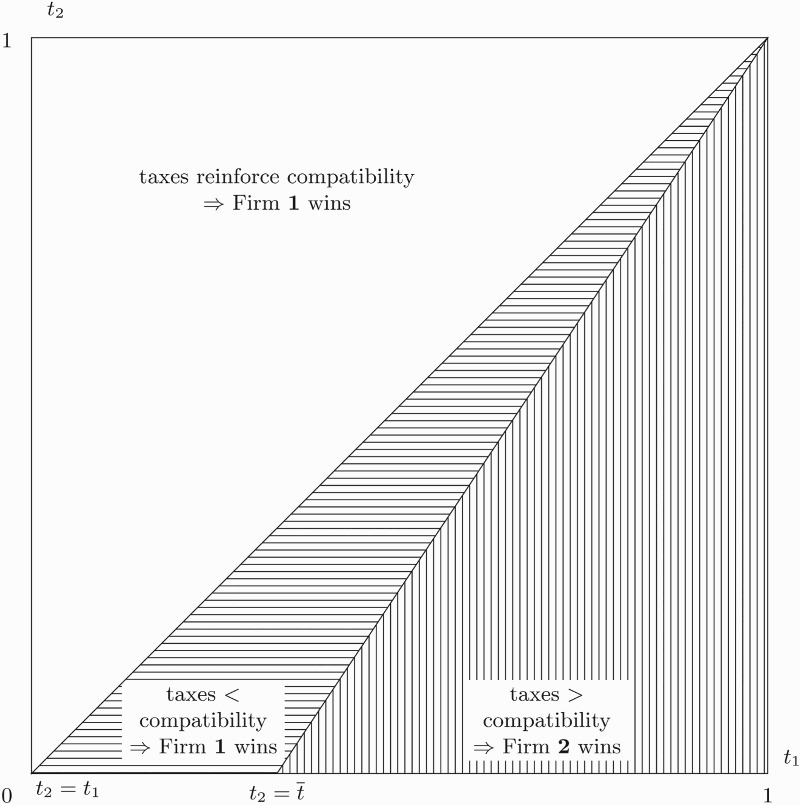
Involved effects and competition outcome with observable effort for different tax rate combinations

Figure 2 illustrates the results from Proposition 4 and depicts all involved effects and the competition outcome depending on different combinations of 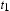 (*x*-axis) and 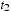 (*y*-axis). The white area shows the situation where 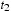 is greater than 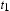. Whenever this is the case, differential taxation has no distorting impact on the competition outcome because the effect of different compatibility is reinforced by the tax effect. However, if 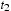 is smaller than 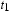, the CEO faces a relatively smaller tax burden when working for firm 2. This effect can moderate (area filled with horizontal lines) or even outweigh (area filled with vertical lines) the disadvantage of different compatibility between the firms. As a result, if 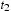 is sufficiently low (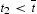) firm 2 is able to win the competition despite lower compatibility.

As the outcome of the competition crucially depends on the cut-off value 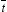, it seems useful to evaluate how this value changes with compatibility and the wage tax rate at firm 1. First, 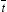 decreases in the compatibility differential among the firms, making it harder for firm 2 to attract the CEO. Intuitively, if there is a high difference in compatibility, the wage tax rate at firm 2 has to be very low in order to still ensure winning the competition. Second, 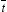 increases in 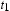. This means that a higher wage tax rate at firm 1 makes it easier for firm 2 to hire the CEO.

In order to derive the salaries offered by each firm, it is again necessary to insert the equilibrium certainty equivalents into Equation (9). As there are two different competition outcomes possible the offered compensation is also dependent on the constituting equilibrium. If firm 1 wins the competition (equilibrium 1), the following values apply:
(14)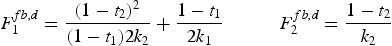 In the equilibrium where firm 2 is successful (equilibrium 2), the offered fixed salaries are as follows:
(15)
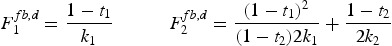



Close inspection of (14) and (15) reveals that the parameters which determine the offered fixed salaries are symmetric for both possible equilibria. This is due to the fact that the equilibrium strategies 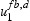 and 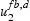 are also symmetric in the same sense.

### 4.2. Equilibrium with Non-Observable Effort

This section considers a more realistic setting where the firms are unable to observe the CEO's behavior directly. In order to derive the outcome of the competition game, it is again necessary to first solve the contracting problem of each firm. In the second-best case the effort exerted by the manager cannot be verified by any of the firms. Therefore, in order to provide working incentives for the CEO, both firms need to make the compensation dependent on the realized cash flow 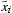 by granting a share 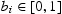 which can be interpreted as a bonus for success. Assuming the first-order approach being valid, the contracting problem of firm *i* can be characterized as follows:
(16)
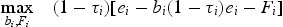

(17)
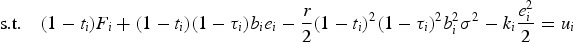

(18)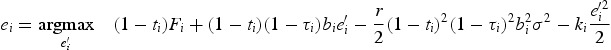 As indicated with Equation (18), in the second-best case the CEO exerts the amount of effort that maximizes the value of the certainty equivalent. Therefore, both firms are bound to this incentive compatibility constraint. As in the first-best case both firms have to offer an amount of certainty equivalent to the agent which is determined by the competition game between the firms. The effort level exerted by the manager conditional on working for firm *i* results from Equation (18) and has the following value:
(19)
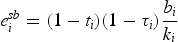
 Anticipating the agent's optimal effort level, firm *i* offers the following compensation contract:
(20)
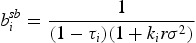

(21)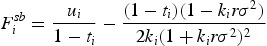 It can be observed immediately that the variable pay firm *i* offers the CEO is independent of 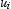. Hence, the bonus payments made to the agent are not influenced by a competition between the two firms. This result is in line with Edmans et al. (2009) who suggest that in a market for talented CEOs, determination of incentive pay can be separated from determination of total pay. Further, the firm includes the corporate tax rate into the bonus parameter in order to compensate the CEO for the after-tax performance measure 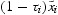. However, the wage tax has no impact on the incentive parameter. As in the first-best case the amount of fixed salary 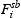 is affected only by the wage tax and is used in order to meet the amount of certainty equivalent 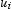 which is determined via the competition game between both firms. Applying the optimal contract parameters, the expected after-tax profit of firm *i* conditional on hiring the CEO takes the following value:
(22)
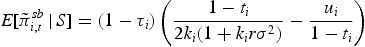



The after-tax profit in Equation (22) is again used to derive the optimal strategies of each firm in the competition game. Similar to Section 4.1, the outcome of the competition game is derived for four different cases arising from different assumptions regarding compatibility and taxation. Propositions 5– 8 present the competition outcome for each case. As a benchmark case, Proposition 5 starts with the situation of equal compatibility and uniform taxation (case 1).

Proposition 5. If the CEO is equally compatible with each firm and taxation is uniform, the competition winner is determined independently from compatibility and taxation. Both firms hire the CEO with equal probability by offering the same amount of certainty equivalent 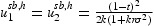. The expected profits of both firms are equal to zero.

Compared to the first-best case, the determination of the competition winner does not change, if both firms are subject to a problem of moral hazard. However, in the second-best case the certainty equivalents offered by each firm become lower by a multiple of 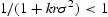. In contrast to standard principal-agent models, the CEO suffers from private information if firms are in a competition. The risk-incentive trade-off within the contracting problem causes an efficiency loss and reduces the expected profit conditional on hiring the CEO (see Equation (22)). The manager extracts all the economic rents from the firms. Therefore, the CEO has also to bear the efficiency loss fully, which translates into a lower certainty equivalent. Proposition 6 presents the competition outcome if the CEO has a higher fit with firm 1 and taxation is uniform (case 2).

Proposition 6. If the CEO is more compatible with firm 1 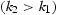 and taxation is uniform, the competition winner depends on compatibility only. Firm 1 always wins the competition and hires the superstar CEO. Both firms offer the same amount of certainty equivalent 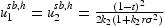. The expected profit of firm 1 is strictly positive whereas the expected profit of firm 2 equals zero.

As in the first-best case, under different compatibility (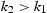) the expected profit of firm 2 conditional on hiring the agent is driven to zero. Firm 1 succeeds in attracting the CEO by offering a certainty equivalent which is slightly higher than 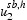 and uniform taxation has no impact on the competition winner.

The equilibrium certainty equivalents in Proposition 6 can be used in order to calculate the fixed salaries which are offered by both competing firms:
(23)
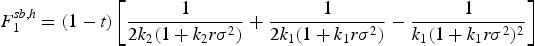

(24)
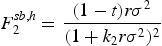



It can be observed that Corollary 1 also holds in a situation with non-observable effort. This means that uniform wage taxes have a negative effect on offered gross fixed salaries, even if the agent has private information about the effort choice.

The remaining part of this section highlights the effect of differential taxation on the competition in the second-best case. Proposition 7 summarizes the outcome of the competition game when the CEO is equally compatible with each firm (case 3).

Proposition 7. If the CEO is equally compatible with each firm and taxation is differential, the competition winner depends on the relation between the wage tax rates 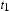 and 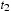 only.

1. If 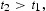 firm 1 wins the competition and hires the superstar CEO. Both firms offer the same amount of certainty equivalent 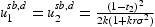. The expected profit of firm 1 is strictly positive whereas the expected profit of firm 2 equals zero.
2. If 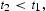 firm 2 wins the competition and hires the superstar CEO. Both firms offer the same amount of certainty equivalent 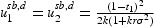. The expected profit of firm 1 equals zero whereas the expected profit of firm 2 is strictly positive.

Assuming equal compatibility, different wage tax rates determine the competition winner exactly in the same way as in the first-best case. The firm facing the lower wage tax rate has competitive advantage and is therefore able to hire the CEO. Finally, the results for a situation of different compatibility and differential taxation (case 4) are presented in Proposition 8.

Proposition 8. If the CEO is more compatible with firm 1 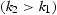 and taxation is differential, the equilibrium outcome of the competition game is dependent on the relation between the compatibility parameters, the wage tax rates 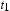 and 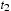 as well as the agent's risk attitude 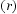 and the firms' business risk 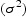. There exist two unique tax-specific cut-off values 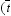 and 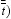 and one unique risk-specific cut-off value 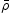 that can be used to determine the competition winner. These cut-off values are defined as follows:

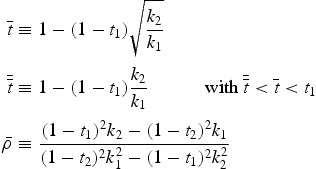

1. If 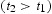 or 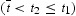 or 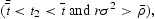 firm 1 wins the competition and hires the superstar CEO. Both firms offer the same amount of certainty equivalent 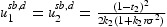. The expected profit of firm 1 is strictly positive whereas the expected profit of firm 2 equals zero.
2. If 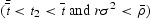 or 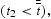 firm 2 wins the competition and hires the superstar CEO. Both firms offer the same amount of certainty equivalent 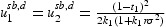. The expected profit of firm 1 equals zero whereas the expected profit of firm 2 is strictly positive.

**Figure 3.**
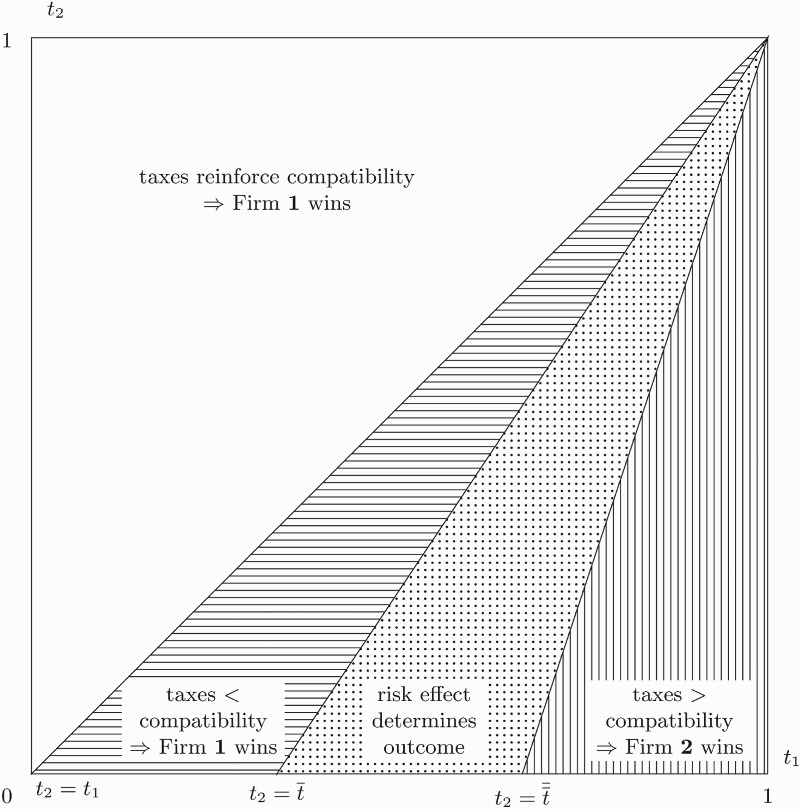
Involved effects and competition outcome with non-observable effort for different tax rate combinations

A comparison between Propositions 4 and 8 reveals that wage taxes affect the competition among firms for the CEO in the second-best case in a slightly different way. This can also be seen in Figure 3 which shows the involved effects and the possible competition outcomes for the case of non-observable effort. Firm 1 is successful in hiring the CEO in equilibrium 1 of Proposition 8. As in the first-best case this equilibrium occurs, if 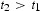 or if 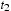 is lower than 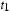 but larger than the cut-off value 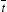. In these cases the tax effect either works in the same direction as the compatibility effect or does not exceed it. However, with non-observable effort and differential taxation, a third effect which can be labeled as ‘risk effect’ affects competition between the two firms. This effect occurs for an intermediate range of 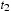 which is represented by the the dotted area in Figure 3. As can be seen from Equation (4), wage taxes reduce the CEO's compensation risk and therefore have a partially moderating effect on the firms' compensation costs. As a result, under differential taxation firm 2 may benefit from a lower individual tax rate, but also suffer from the risk induced effect if risk is sufficiently important relative to compatibility and taxes (*r* and/or 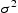 are sufficiently high). Therefore, whenever 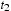 is between 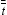 and 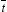, firm 1 is able to win the competition for the CEO, if the risk effect dominates the trade-off between taxes and compatibility (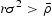). On the other hand, if the risk effect is sufficiently small and hence lacks importance, firm 2 is also able to hire the CEO whenever 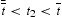 (equal to the first-best case). It should be noted that the technically necessary assumption of normally distributed payments might also imply an overstatement of the risk premium in Equation (4) which is demanded by the CEO. Therefore, in cases of limited liability or where immediate tax reimbursements are not applicable, it could be that the risk effect becomes too weak in order to have an impact on the outcome of the competition. However, as already mentioned in the model description there exist also real world scenarios where these restrictions do not occur for single periods. Finally, in the case of 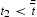 the tax benefit dominates any other effect and firm 2 always wins the competition for the CEO.

The resulting fixed salaries are dependent on the constituting equilibrium. If firm 1 is able to hire the agent, the following payments are offered:
(25)
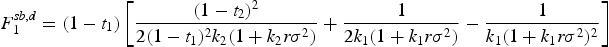

(26)
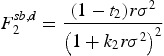
 As in the first-best case, the payments are symmetrical for equilibrium 2 of Proposition 8 and therefore not presented here.

### 4.3. Comparative Statics

This section provides an intuition for the effects of marginal tax rate changes on the equilibrium parameters presented in Proposition 8. Due to the symmetry of equilibrium 1 and 2 with respect to the equilibrium parameters the comparative statics are derived in a generalized way with firm *w* representing the competition winning firm which is hiring the CEO, and firm *l* representing the inferior competitor (loser). First, it should be noted that even though the corporate tax has no impact on the competition outcome, it nevertheless determines the level of the offered bonus parameter (see Equation (20)). As the after-tax bonus parameter of each firm explicitly corrects for the tax-induced reduction of the performance measure someone can expect to observe higher performance sensitivity at firms which are located in countries with higher corporate tax rates, if these firms use after-tax performance measures.

Next, the impact of wage taxes on the fixed salary offered by the inferior competitor *l* and on the expected utility the CEO receives in equilibrium 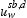 is presented below:
(27)
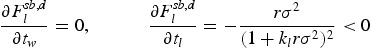

(28)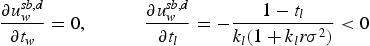 As indicated in Equations  (27) and (28) the offered fixed salary of the inferior firm *l* and the expected utility received by the CEO are insensitive to marginal changes of the wage tax rate applied at firm *w* and are declining in the wage tax rate applied at firm *l*. The intuition behind these observations can be explained by the same effects driving the result in Corollary 1. The wage tax applied at firm *l* reduces the optimal amount of effort exerted by the CEO. As a result the CEO's potential economic rent declines, which in turn reduces both the expected utility and the offered fixed salary of firm *l*. However, as in equilibrium the CEO is (nearly) held at reservation utility (the utility from working for firm *l*), marginal changes of 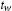 do not imply any effects on the expected utility or the salary offered by firm *l*. Further, the above described effects partially explain the effects of tax rate changes on the fixed salary offered by firm *w*:
(29)
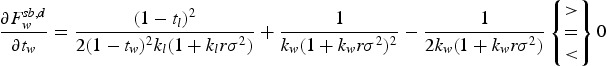

(30)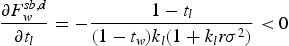 Equation (30) shows that an increase of 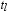 leads to a decline of the fixed salary offered by the successful firm *w*. This indirect effect occurs because a higher 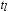 reduces the expected utility received by the CEO in equilibrium (see Equation (28)), and enables firm *w* to lower the offered fixed salary without losing the CEO. However, the tax rate applied at the successful competitor *w* has an ambiguous effect on the fixed salary offered to the CEO.^15^ Due to the complex structure of Equation (29), deriving necessary conditions with respect to the sign of the partial derivative does not yield very meaningful expressions. Nevertheless, it is possible to observe that the wage tax rate 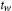 has a positive effect on the offered fixed salary 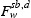 whenever the first two addends exceed the third one. This can be summarized in the following corollary:

Corollary 2. The fixed salary offered by the competition winning firm *w* is more likely to increase in the wage tax rate 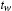 if

1. the compatibility with firm *l* is high 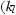 is small
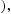

2. the compatibility with firm *w* is low 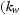 is high
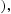

3. the wage tax rate at firm *l* is low,
4. the wage tax rate at firm *w* is high.

As all the situations in Corollary 2 seem to be favorable for the losing firm, a positive effect of the wage tax on the salary should occur when there has been a narrow victory for firm *w* indicating strong competition. Intuitively, if the competition among the two firms is very severe (e.g. neither effect dominates the others by very much), an increase of the wage tax provides the successful firm with a considerable additional burden and the attraction of the agent can only be maintained by increasing the offered fixed salary. This result could also help to interpret the results of empirical papers that investigate the impact of wage tax rate changes on observed gross compensation in international labor markets. A positive relation between observed gross compensation and 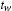 could indicate that competition among firms for highly skilled employees is very severe because the difference between the competing firms is relatively low. If competition is severe, the bargaining power of the employee increases and improves the ability to shift an additional tax burden to the firm.

Finally, effects of marginal tax rate changes on the expected profit of the successful competitor *w* are investigated below. The expected profit of the successful competitor can be calculated by inserting the equilibrium certainty equivalent derived in Proposition 8 into Equation (22):
(31)
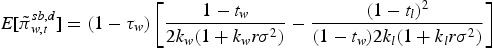



Taking the partial derivative of (31) with respect to the wage tax rates yields the following:
(32)
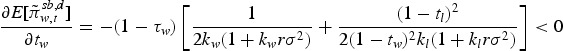

(33)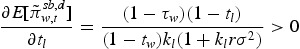 Despite the ambiguous effect of 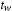 on the fixed salary offered by the successful firm *w*, an increase of 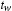 always has a negative impact on the expected profit. This effect can be explained by a joint examination of Equation (22) with Equation (28). On one hand, 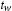 does not change firm *w*'s optimal strategy 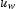 in the competition game. On the other hand, 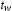 reduces the expected profit due to a lower optimal amount of effort and the additional wage costs incurred by the difference between gross and net wages. However, an increase of the tax rate the CEO would face when working for the inferior firm *l* has an unambiguous positive effect on the successful firm's expected profit, because it helps to lower the needed certainty equivalent offer 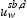 which still ensures attraction of the agent.

## 5. Conclusion

This paper analyzes the effects of taxation on the competition between two firms for a unique and highly skilled CEO. By using a partial equilibrium model of moral hazard it illustrates the possible impact of uniform and differential taxation on the outcome of competition as well as the optimal contracts offered by both firms. The paper showed that the competition winner crucially depends on the CEO's compatibility with both firms. Further, the competition winner is insensitive to uniform taxation of profits and wages. However, it was possible to derive the novel result that offered gross fixed salaries decline with uniform wage taxes in a local competition setting. The main explanation for this observation can be found in the CEO's ability to extract a considerable amount of economic rents from both firms due to being unique. As a wage tax decreases these rents, it exerts also a softening effect on the competition among the firms, which in turn translates into reduced gross fixed salaries.

A key finding of this paper is that different wage tax rates have a substantial impact on the outcome of the competition between two firms. Sufficiently large tax rate differentials can offset the effect of compatibility and hence lead to situations where the less compatible firm is able to hire the CEO. However, this tax effect is moderated in a situation of non-observable effort with sufficiently risky cash flows or a sufficiently risk averse CEO. Further, the paper showed that different corporate tax rates have no impact on the competition outcome. Nevertheless, they translate into different (gross) incentive parameters offered by the firms because of the tax-reduced performance measure. Additionally, the paper provides some intuitions on the effects of marginal changes of all wage tax rates involved. An increase of the tax rate applied at the successful firm has a negative effect on this firm's expected profit, but does not change the expected utility received by the CEO. An increase of the tax rate where the inferior firm is located reduces the expected utility received by the CEO and increases the expected profit of the successful firm due to lowered compensation costs.

It should be possible to draw some important conclusions for the tax regulator and empirical tax research from this investigation. In the public discussion about excessive compensation of top level managers, regulation or redistribution through taxation is demanded very frequently. The paper showed, however, that raising wage tax rates unilaterally might imply a substantial competitive disadvantage for attracting highly skilled human resources in an international labor market. This adverse effect depends crucially on the characteristics of the involved firms, and it occurs only if the resulting tax rate differential is sufficiently high. Additionally, the paper illustrates that a unilateral increase of the wage tax rate has an ambiguous effect on the observed gross wage levels. The model predicts that a positive association is more likely if competition between firms is very severe. This result could also serve as an explanation for the mixed results of empirical studies in this area as there might exist a considerable variation with respect to severity of competition.

Finally, the analysis is subject to several limitations which should be discussed very briefly. First, it should be noted that the partial equilibrium model applied in this paper might be silent about effects that can only be observed in a general equilibrium. Even though the model seems to be quite robust to an increasing number of competing firms, a transfer of the attained results to a labor market with an excess supply of talented CEOs would need further justification. Second, in the presented model private information of the CEO is limited to the amount of effort exerted for the hiring firm. However, one can expect that the agent's compatibility with either firm can be an additional source of information asymmetry between the CEO and both firms in practice.^16^ As this problem would not only affect the firms' optimal contracting solution, but also the outcome of competition between the firms, an analysis of this situation could enrich our understanding of this special kind of labor market and should therefore be considered for future research. Third, the investigation illustrated that the firms' business risk and the CEO's risk attitude can play a major role for the competition among firms in the case of non-observable effort with different tax rates. For this reason it would be interesting to see whether a departure of the simplifying assumption of equal business risks could cause further frictions with respect to the pre- and after-tax outcome of the competition. As higher risk is another source of competitive disadvantage, one can imagine interesting situations which would generate an additional trade-off between risk and compatibility, even if taxes are equal or negligible.

## Footnotes

1 Excluding Facebook as an extreme outlier still yields a ratio of 295.9-to-1 for the year of 2013.

2 Throughout the paper, the terms ‘CEO’, ‘manager’, and ‘agent’ are used interchangeably.

3 Other trends include for instance relatively high insensitivity of CEO compensation to firm performance and reward for luck. Edmans and Gabaix (2009) provide a brief review about these trends and discuss a series of potential explanations for them.

4 Section 162(m) IRC has been introduced in 1993. It limits deductions for non-performance-based compensation of the CEO and the four highest compensated officers to $1 million per year.

5 Subscript *t* indicates the corresponding after-tax expressions for all variables.

6 It should be noted that the agent's gross compensation  is based on the after-tax cash-flow . Other possible performance measures could be the firm's after-tax profit (e.g. Ewert and Niemann, 2012) or profit before taxes. In a recent study, Gaertner (2014) shows that nearly 61% of his sample firms use after-tax incentives. This result indicates heterogeneity across firms with respect to the use of after-tax performance measures.

7 Superscript *fb* indicates the case of observable effort whereas superscript *sb* denotes the case of non-observable effort.

8 It should be noted that the optimal level of effort decreases in the wage tax, due to the substitution of income with leisure time. With the specified utility function, wage taxes do not perform an income effect as proposed by Atkinson and Stiglitz (1980) because there does not exist an additional source of income. However, according to Kuhn (2015) the absence of an income effect could also result from an individual's ability to shift income and labor supply from one period to another (e.g. through perfect capital markets) which serves as a possible justification for the model in use.

9 All equilibrium values associated with the situation of uniform (homogeneous) taxation are denoted by a superscript *h*.

10 From a formal perspective, it should be noted that firm 1 has to overbid the offer of firm 2 by a a small amount  in order to hire the CEO with certainty. However, throughout this paper this technical necessity is neglected because *ϵ* can be very close to zero and including it does not yield any further insights (see also the proof in the Appendix). Alternatively, someone could also simply assume, that an indifferent CEO chooses always the firm earning a higher expected profit.

11 All equilibrium values associated with the situation of differential taxation a denoted by a superscript *d*.The proofs are similar to those in Proposition 1 and 2 and therefore omitted for brevity. This is also done for the remaining propositions in this paper for the same reason.

12 The case of  is ignored here, as it yields the same result as with equal compatibility and uniform taxation presented in Proposition 1.

13 It should be noted that this result is based on an assumption regarding the after-tax expected profits from hiring an ordinary manager (the firms' outside opportunity): It is assumed that these after-tax expected profits become equal to a unified market ‘return’ due to competition. (That is, ). Additionally differences in corporate tax rates might become relevant, if parts of the CEO's compensation are non-deductible for the firm. Halperin et al. (2001) and Göx (2008) provide an extensive analysis of this issue in the context without competition for CEOs.

14 The case of  is ignored here, as it yields the same result as with equal compatibility and uniform taxation indicated in Proposition 1.

15 With observable effort, the fixed salary offered by firm *w* is strictly decreasing in the wage tax rate  because . Therefore, it is possible that private information and the implied risk effect does not only have an impact on the competition winner but also on marginal effects of the wage tax rate.

16 Bénabou and Tirole (2016) investigate such a scenario without considering any issues of international taxation.

## Appendix

### Proofs

Proof of Proposition 1. Proof of Proposition 1. In order to prove that the determination of the competition winner is independent from compatibility and taxation it is first necessary to derive and prove the equilibrium strategies for both firms. In equilibrium, the expected profit of each firm is at least as high as the expected profit from hiring an ordinary CEO (). Therefore,  with . In order to prove that in equilibrium both firms offer the same certainty equivalent suppose first that . In this case the CEO would be hired by firm 2 and the expected profit of firm 1 equals zero. However, firm 1 could increase its expected profit by raising the offered certainty equivalent to  and thereby attracting the CEO which contradicts  being an equilibrium. Second, suppose that . This configuration can not be an equilibrium as well since firm 2 has the opportunity to strictly increase its profit by decreasing the offered certainty equivalent by a small amount. Therefore, in equilibrium . Assume now that . In this situation both firms have an incentive to deviate by offering a certainty equivalent which is slightly higher (some small amount ) in order to increase their expected profit. Hence, the equilibrium constituted by  is the only Nash equilibrium. Inserting the equilibrium strategies into the expected after-tax profit conditional on hiring the CEO in (11) yields an expected profit of zero for each firm. Finally, it can be proven that that the competition winner is determined independently from compatibility and taxation. As the strategies of both firms are exactly identical in equilibrium, the CEO is held indifferent between the two firms and randomizes with equal probability. The equality of equilibrium strategies and the randomization does not change for any variation of *k* or *t*. This is sufficient to prove that equal compatibility and uniform taxation are not determining the competition winner.

Proof of Proposition 2 Proof of Proposition 2. In order to prove that the determination of the competition winner depends on compatibility only it is first necessary to derive and prove the equilibrium strategies for both firms. When the CEO's compatibility is different () by the same arguments as for Proposition 1 there cannot exist an equilibrium with . Further, in equilibrium firm 2 sets  and has no incentive to deviate unilaterally in any direction because it cannot increase its expected profit. However, firm 1 is able to earn some positive expected profit by setting . If  the CEO randomizes between both firms and the expected profit of firm 1 equals . By increasing the offered certainty equivalent by a very small amount  firm 1 could hire the CEO with certainty and earn an expected profit equal to

(A1)

Assume that *ϵ* is the smallest currency unit available and fulfills that . Then, in equilibrium firm 1 offers  because this yields the higher expected profit. Further assume that *ϵ* is very close to zero and hence can be neglected. In the limit () firm 1 is always able to hire the CEO with certainty by offering the same certainty equivalent . The expected profit of firm 2 is zero because it has to hire an ordinary manager. The expected profit of firm 1 equals

(A2)

With  this expected profit is strictly positive. This competition equilibrium and firm 1 being the competition winner holds as long as . Therefore, compatibility is the only determinant of the competition winner.

Proof of Corollary 1 Proof of Corollary 1. The first derivatives of the fixed compensation offered by the firms in Equation (13) are as follows:

(A3)

(A4)

With , these first derivatives are unambiguously negative.
